# Supplementary material for: Male-predominant galanin mediates androgen-dependent aggressive chases in medaka
Source: eLife. 2020 Aug 12;9:e59470. doi: 10.7554/eLife.59470 (PMC7423395; doi:10.7554/eLife.59470)
Supplement: Supplementary file 1. [file elife-59470-supp1.docx]

Supplementary file 1. Abbreviations of medaka brain regions and nuclei.

| abbreviation | full name | location |
| --- | --- | --- |
| brain region |  |  |
| Cb | cerebellum |  |
| dcTel | dorsocaudal part of the telencephalon |  |
| Hyp | hypothalamus |  |
| MO | medulla oblongata |  |
| MT | midbrain tegmentum |  |
| OB | olfactory bulb |  |
| OpN | optic nerve |  |
| OT | optic tectum |  |
| Pit | pituitary |  |
| Tel | telencephalon |  |
| Th | thalamus |  |
| vrTel | ventrorostral part of the telencephalon |  |
| brain nucleus |  |  |
| aPMp | anterior part of PMp | preoptic area |
| CbSP | Purkinje cell layer of the corpus cerebellum | cerebellum |
| CP | central posterior nucleus (thalamus) | thalamus |
| Dc | central nucleus of the dorsal telencephalic area | dorsal telencephalon |
| Dd | dorsal nucleus of the dorsal telencephalic area | dorsal telencephalon |
| Dl | lateral nucleus of the dorsal telencephalic area | dorsal telencephalon |
| Dm | medial nucleus of the dorsal telencephalic area | dorsal telencephalon |
| DP | dorsal posterior nucleus (thalamus) | thalamus |
| Dp | posterior nucleus of the dorsal telencephalic area | dorsal telencephalon |
| gc | central gray (brain stem) | brain stem |
| IQ | inferior oblique of the oculomotor nerve nucleus | midbrain tegmentum |
| IR | inferior rectus of the oculomotor nerve nucleus | midbrain tegmentum |
| is | isthmus nucleus | midbrain tegmentum |
| LV | lateral valvular nucleus | midbrain tegmentum |
| LX | vagal lobe | medulla oblongata |
| MR | medial rectus of the oculomotor nerve nucleus | midbrain tegmentum |
| NAT | anterior tuberal nucleus | hypothalamus |
| NDTL | diffuse nucleus of the lateral torus | hypothalamus |
| NFS | solitary fascicular nucleus | medulla oblongata |
| NPT | posterior tuberal nucleus | hypothalamus |
| NRL | lateral recess nucleus | hypothalamus |
| NVT | ventral tuberal nucleus | hypothalamus |
| PGZ3 | periventricular gray zone (layer 3) | optic tectum |
| PMm | magnocellular portion of the magnocellular preoptic nucleus | preoptic area |
| PMp | parvocellular portion of the magnocellular preoptic nucleus | preoptic area |
| PPa | anterior parvocellular preoptic nucleus | preoptic area |
| pPMp | posterior part of PMp | preoptic area |
| PPp | posterior parvocellular preoptic nucleus | preoptic area |
| RI | inferior reticular nucleus | medulla oblongata |
| RM | medial reticular nucleus | medulla oblongata |
| TS | semicircular torus | midbrain tegmentum |
| VM | ventromedial nucleus (thalamus) | thalamus |
| Vp | posterior nucleus of the ventral telencephalic area | ventral telencephalon |
| Vs | supracommissural nucleus of the ventral telencephalic area | ventral telencephalon |
| Vv | ventral nucleus of the ventral telencephalic area | ventral telencephalon |
