# Supplementary material for: Male-predominant galanin mediates androgen-dependent aggressive chases in medaka
Source: eLife. 2020 Aug 12;9:e59470. doi: 10.7554/eLife.59470 (PMC7423395; doi:10.7554/eLife.59470)
Supplement: Supplementary file 2. [file elife-59470-supp2.docx]

Supplementary file 2. Species names and GenBank accession numbers of the protein sequences used in this study.

| protein | species | accession number |
| --- | --- | --- |
| GAL | human (*Homo sapiens*) | NP_057057 |
| GAL | bovine (*Bos taurus*) | NP_776339 |
| GAL | mouse (*Mus musculus*) | NP_034383 |
| GAL | rat (*Rattus norvegicus*) | NP_150240 |
| Gal | chicken (*Gallus gallus*) | NP_001138861 |
| Gal | stickleback (*Gasterosteus aculeatus*) | ALD51505 |
| Gal | zebrafish (*Danio rerio*) | AJG05990 |
| GALP | human (*Homo sapiens*) | NP_149097 |
| GALP | mouse (*Mus musculus*) | NP_821171 |
| SPX | human (*Homo sapiens*) | NP_085049 |
| SPX | mouse (*Mus musculus*) | NP_001229274 |
| GALR1 | human (*Homo sapiens*) | NP_001471 |
| GALR1 | bovine (*Bos taurus*) | NP_001179170 |
| GALR1 | mouse (*Mus musculus*) | NP_032108 |
| GALR1 | rat (*Rattus norvegicus*) | NP_037090 |
| Galr1 | chicken (*Gallus gallus*) | NP_001121534 |
| Galr1 | medaka (*Oryzias latipes*) | XP_004086705 |
| Galr1a | seabass (*Dicentrarchus labrax*) | AIU47913 |
| Galr1b | seabass (*Dicentrarchus labrax*) | AIU47914 |
| GALR2 | human (*Homo sapiens*) | NP_003848 |
| GALR2 | bovine (*Bos taurus*) | NP_001098480 |
| GALR2 | mouse (*Mus musculus*) | NP_034384 |
| GALR2 | rat (*Rattus norvegicus*) | NP_062045 |
| Galr2 | chicken (*Gallus gallus*) | NP_001121535 |
| Galr2a | seabass (*Dicentrarchus labrax*) | AIU47916 |
| Galr2b | seabass (*Dicentrarchus labrax*) | AIU47915 |
| GALR3 | human (*Homo sapiens*) | NP_003605 |
| GALR3 | mouse (*Mus musculus*) | NP_056553 |
| GALR3 | rat (*Rattus norvegicus*) | NP_062046 |
| Galr3 | chicken (*Gallus gallus*) | NP_001124057 |
| AlstR | *Drosophila melanogaster* | NP_524700 |
| Npr-9 | *Caenorhabditis elegans* | NP_509896 |
