# Supplementary material for: Male-predominant galanin mediates androgen-dependent aggressive chases in medaka
Source: eLife. 2020 Aug 12;9:e59470. doi: 10.7554/eLife.59470 (PMC7423395; doi:10.7554/eLife.59470)
Supplement: Supplementary file 3. [file elife-59470-supp3.docx]

Supplementary file 3. Primers used in this study.

| target | direction | purpose | sequence (5' to 3') |
| --- | --- | --- | --- |
| *gal* | forward | real-time PCR | ATAGACGGACACAGGACA |
| *gal* | reverse | real-time PCR | TAGGACAGGAAATCCACCAC |
| *actb* | forward | real-time PCR | CCCCACCCAAAGTTTAG |
| *actb* | reverse | real-time PCR | CAACGATGGAGGGAAAGACA |
| *gal* | forward | genotyping of knockouts (PCR on genomic DNA) | CTGGCCGCTCCGCTCACTCA |
| *gal* | reverse | genotyping of knockouts (PCR on genomic DNA) | AAGGCTGGCATGTTCCCTGATCT |
| *gal* | forward | genotyping of knockouts (cycle sequence) | GGCTCCAGCTCATCCGCTTCT |
